# Pelvic Inflammatory Disease: Possible Catches and Correct Management in Young Women

**DOI:** 10.1155/2018/5831029

**Published:** 2018-07-11

**Authors:** Chiara Di Tucci, Daniele Di Mascio, Michele Carlo Schiavi, Giorgia Perniola, Ludovico Muzii, Pierluigi Benedetti Panici

**Affiliations:** Department of Gynecological and Obstetric Sciences, and Urological Sciences, University of Rome “Sapienza”, Umberto I Hospital, Rome, Italy

## Abstract

The incidence of adnexal masses increases exponentially with age and the most frequent causes in young women are physiologic cysts and pelvic abscesses with pelvic inflammatory disease (PID). Clinical examination can direct physicians to an appropriate management of adnexal mass, but the role of transvaginal ultrasound is crucial for diagnosis and treatment decision, even if it sometimes can be misleading, especially in young women. Ca 125, blood count, and CRP are useful to clarify suspected etiology of a pelvic mass, but specificity and positive predictive value are low because elevation of laboratory tests may occur in several benign conditions. In our work we present four cases of suspected pelvic masses. Despite guidelines for management of PID, the right timing to switch to surgical therapy is not clear. Therefore, the treatment decision should be based on a careful evaluation of various parameters such as signs symptoms and above all age. Moreover, we believe that, for a correct diagnosis and for the best fertility sparing treatment, it is also extremely important to refer to a gynecological oncology unit with an expert surgeon.

## 1. Introduction

The incidence of adnexal masses increases exponentially with age, and in 25-40-year-old women the prevalence of an adnexal lesion is reported to be about 7.8% [1]. The incidence of PID is particularly difficult to evaluate, because of high rates of subclinical PID, increasing rates of outpatient diagnosis, and inaccuracies in the diagnosis [2].

The most frequent benign gynecological causes are generally physiologic cyst [3], while the most frequent malignant causes in adolescent and young women are borderline ovarian tumors (BOT) and sex cord-stromal or germ cell tumors in 43% and 23% of women <35 years old [4]. Therefore, a right diagnosis and management of adnexal masses in these patients are extremely important and should be directed to identify the cause and preserve fertility as much as possible. Clinical examination based on symptoms and signs and laboratory tests may help physicians, but imaging significantly increases the accuracy of differential diagnosis [5]. Transvaginal ultrasound is the most commonly used imaging technique for preoperative characterization of any adnexal mass, with a sensitivity of 93% and a specificity of 81% when adopting the International Ovarian Tumor Analysis (IOTA) “Simple Rules” classification system [6]. Despite these excellent accuracy rates, when laparoscopic surgery is limited to women with cysts that appear benign, the rate of unexpected malignancy is 0-2.5% [7]. Furthermore, IOTA criteria cannot replace the gynecologist's skills, which are essential to improve overall survival rate, trying to preserve fertility at the same time. In this work, we present four cases of women with pelvic mass and the subsequent management, better describing the issues mentioned above.

## 2. Case One

**Figure 1:**
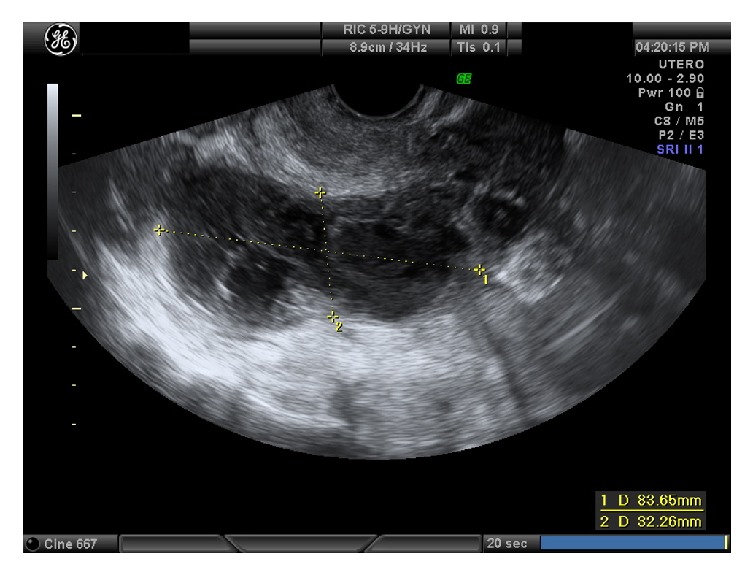
Case one: right adnexal mass.

A 31-year-old Italian patient came to our attention for pelvic pain in December 2016. She had no known previous history of sexually transmitted infections (STIs) nor known chronic disease under pharmacological treatment. She did not practice any contraceptive method and currently had a single sex partner with whom she had unprotected sex, trying to get pregnant. Last Pap smear (October 2016) reported mild inflammation and Doderlein's cytolysis. She had a regular 28-day menstrual cycle. In August 2015, she had been already admitted in another hospital for pelvic pain and she was treated with antibiotic therapy for a suspected pelvic inflammatory disease (PID). In October 2015 she had positive cervicovaginal swabs for Candida Albicans. In March 2016 she had negative Ca125 and AFP blood test (17.7 u/mL and 1.6 ng/mL, respectively). On physical examination, she referred pain in abdominal lower quadrants. During gynecological examination, we found leucorrhoea after the introduction of the speculum. She had low grade fever and blood tests showed hemoglobin level: 13.0 g/dL; total white blood cells: 4.63x10^9^/L; neutrophils: 44.6%; platelets: 231x10^9^/L and C-reactive protein (CRP) was positive (4 mg/dL; 0-0.5 mg/dL). Transvaginal ultrasound performed at the admission showed a normal sized uterus and a multilocular, solid, thick-walled structure with internal incomplete septa and color score 1 in both adnexal regions (65x58mm to the left and 83x32mm to the right) (Figure 1). Fluid and “sliding sign” were shown in the Pouch of Douglas. At laparoscopy, two voluminous adnexal masses, measuring about 7 cm, with exophytic vegetation and superficial vascularization, were seen bilaterally. Extemporaneous histological examination of biopsy showed the presence of serous BOT. A bilateral cystectomy and peritoneal staging were performed. Two weeks later, the final histological examination confirmed the previous diagnosis.

## 3. Case Two

A 24-year-old Italian patient was admitted to our hospital in February 2017 for severe pelvic pain progressively increasing in severity, bloating, and constipation. She had three sex partners and she did not practice any contraception. Last Pap smear (December 2016) was negative. She had regular menstrual cycle, with an increased flow (Heavy Menstrual Bleeding) and dysmenorrhea in the last three months. On physical examination, she presented abdominal pain, especially in lower quadrants. During gynecological examination, we did not find any pathological vaginal and cervical lesion and leucorrhoea was not present, but a pelvic neoformation of about 10 cm increasing in consistency was appreciated with the bimanual pelvic and digital rectal examination. She had no fever and inflammatory markers were normal (total white blood cells: 4.63x109/L; neutrophils: 44.6%; CRP: negative). Transvaginal ultrasound showed a normal uterus and a multilocular solid mass of 90x30x40 mm with internal incomplete septa and color score 2 in left adnexal region. AFP, bHCG, and HE4 were negative, but Ca125 level was 834 u/mL, with a twofold increase in 15 days (400 u/mL at the beginning of February). The ultrasound examination skewed towards a pelvic inflammation, but we opted for an urgency laparoscopy, because of the woman's young age, pain, and the sudden Ca125 increase. After optical introduction, hydrosalpinx and widespread signs of pelviperitonitis were found. Drainage with removal of pus and multiple biopsies were performed. Histologic samples showed signs of flogosis. The patient was placed on doxycycline 100 mg twice a day for 7 days according to the Center for Disease Control and Prevention (CDC) guidelines for the management of PID [7]. She was educated on the method and the importance of preventing sexually transmitted diseases (STDs) and advised to treat her sexual partner with the same treatment.

## 4. Case Three

A 32-year-old Italian patient came to our attention for pelvic pain and fever in April 2017. She had been already admitted to our department for pelvic pain and she was treated with antibiotic therapy for a suspected PID. She had low grade fever and blood tests showed hemoglobin level: 11.8 g/dL; total white blood cells: 12.26∖x10^9^/L; neutrophils: 92.2%; platelets: 258x10^9^/L; CRP: 8.85 mg/dL (0-0.5 mg/dL). She had no known previous history of sexually transmitted infections (STIs). Last Pap smear (2016) was referred to be negative. One week after antibiotic therapy, we observed a slight decrease of CRP and remission of temperature, but also an increased pain in abdominal lower quadrants. Transvaginal ultrasound performed at the admission showed a normal uterus and, in the left adnexal region, a dilated, low-vascularized tube (43x29mm) attached to the left ovary (57x54x44mm) was shown. Right ovary was normal. No fluid was found in the Pouch of Douglas. The patient underwent diagnostic and operative laparoscopy: after having introduced the optic trocar, a mass with some colliquative areas in the left tubal corner of the uterus, measuring about 4 cm, was seen. The extemporaneous histological examination showed the presence of endometrial stromal sarcoma. Two weeks later, the final histological examination confirmed the previous diagnosis.

## 5. Case Four

In January 2018, a 22-year-old Italian patient came to our attention for pelvic pain associated with dysuria and pollakiuria, without fever or vaginal discharges. She had previous history of right ovariectomy for dermoid cyst in 2009.

**Figure 2:**
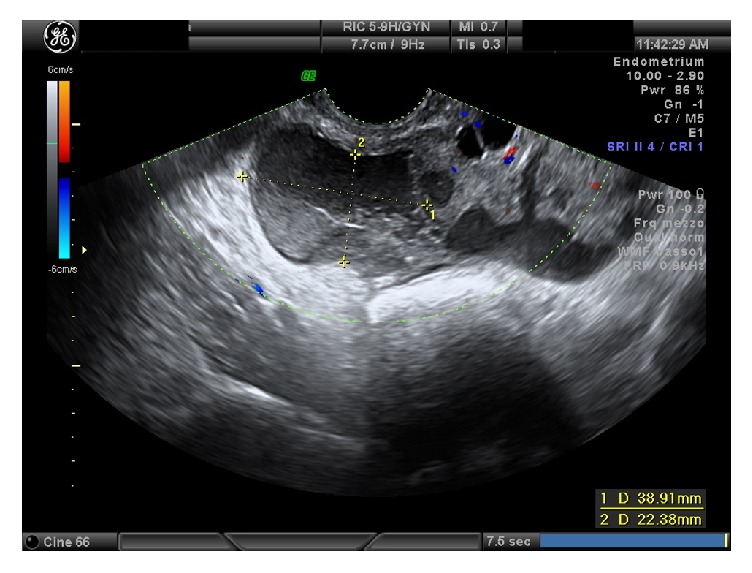
Case four: left adnexal mass (1).

**Figure 3:**
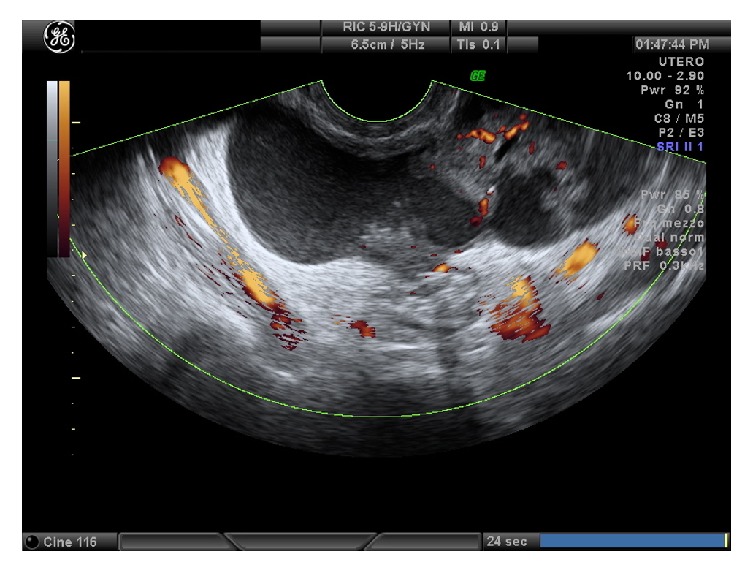
Case four: left adnexal mass (2).

Transvaginal ultrasound performed at the admission showed a normal uterus and a ground glass area (39x22 mm) in left adnexal region and a second dilated, low-vascularized, ground glass area, (80x28 mm) in the left ovary (20x10x15 mm) (Figures 2 and 3). No fluid was found in the Pouch of Douglas. White blood cells were normal and CEA, CA19.9, and AFP were normal, but CRP was 10.800 microg/L, HE4 was 84.1, and Ca125 level was 463.6 u/mL. MRI described the presence of a formation with fluid content in the ovarian and paraovarian region with oval morphology of 35x15 mm; nearly MRI also revealed the presence of a formation with elongated morphology from the left ovary to the contralateral region adnexal. Antibiotic therapy was started, but then she undergone surgery, due to increase of CRP and pelvic pain persistence. After the introduction of the optic trocar, right salpinx presented with ectasia (hydrosalpinx), attached to the intestinal loops. Left adnexa presented an ovarian abscess. After having detected the ureters, the right tube was cleared. Drainage of the left adnexa abscess and lysis of adhesions were performed.

## 6. Discussion

Gynecological adnexal masses may origin from uterus, tubes, or ovaries and the goal of the evaluation is to tell benign conditions from malignant ones. During reproductive age, adnexal masses may present as symptoms that can guide the differential diagnosis, but sometimes symptoms can also lead to a misdiagnosis. For this reason, imaging may be helpful for diagnostic evaluation and treatment decision. Transvaginal ultrasound is the recommended imaging modality for a suspected or an incidentally identified pelvic mass, with good patient tolerability and cost-effectiveness [8]. Nevertheless, ultrasound examination sometimes can be misleading in young women. In our first case, clinical symptoms, bilateral involvement, and inflammatory markers together with ultrasound examination led us to assume a diagnosis of PID, but we opted for surgery because of persistent pain despite medical treatment. Ca125, blood count, and CRP are useful to clarify suspected etiology of a pelvic mass and should be used to assess the possibility of malignancy. Nevertheless, specificity and positive predictive value of Ca125 levels are consistently higher in postmenopausal women compared with premenopausal women [9] and elevation of Ca125 levels in premenopausal women may occur in several conditions including fibroids, endometriosis, adenomyosis, pelvic infection, and nongynecologic diseases [10–12]. In our second case, the twofold Ca125 levels led us to hypothesize malignancy, even if women with benign conditions such as III/IV stage endometriosis can have Ca125 level elevation of 1,000 units/mL or greater, but ultrasound imaging directed us towards the hypothesis of a suspected infection. CDC guidelines suggest medical treatment as the first-line treatment of PID, but the patient's young age and the worsening pelvic pain led us to opting for surgical treatment to exclude malignant lesions and to try to preserve fertility [8]. There is some evidence for CRP to be an interesting marker of tumor bulk when associated with the clinical-pathological parameters, FIGO tumor stage, and postoperative residual tumor mass. In fact, elevated CRP serum levels independently predicted the presence of borderline and epithelium ovarian tumor in patients with suspicious adnexal masses and were reported to be of additional value to Ca125 in the preoperative differential diagnosis of adnexal masses [13]. In our first case too, the woman showed an elevated value of CRP. Young women with pelvic pain should be also screened for sexual transmitted disease to rule out infections that could lead to infertility, as recurrent PID is associated with an almost twofold increase in infertility [14]. In case of no response to medical therapy, patients should be directed to surgery to preserve fertility and to rule out the possibility of unexpected ovarian malignancy [7]. In our opinion, the presence of a gynecologic surgeon is mandatory, as it is significantly more likely to result in ovarian conservation [15], and the role of a gynecologic oncologist is associated with improved overall survival, in case of malignancies [16].

**Figure 4:**
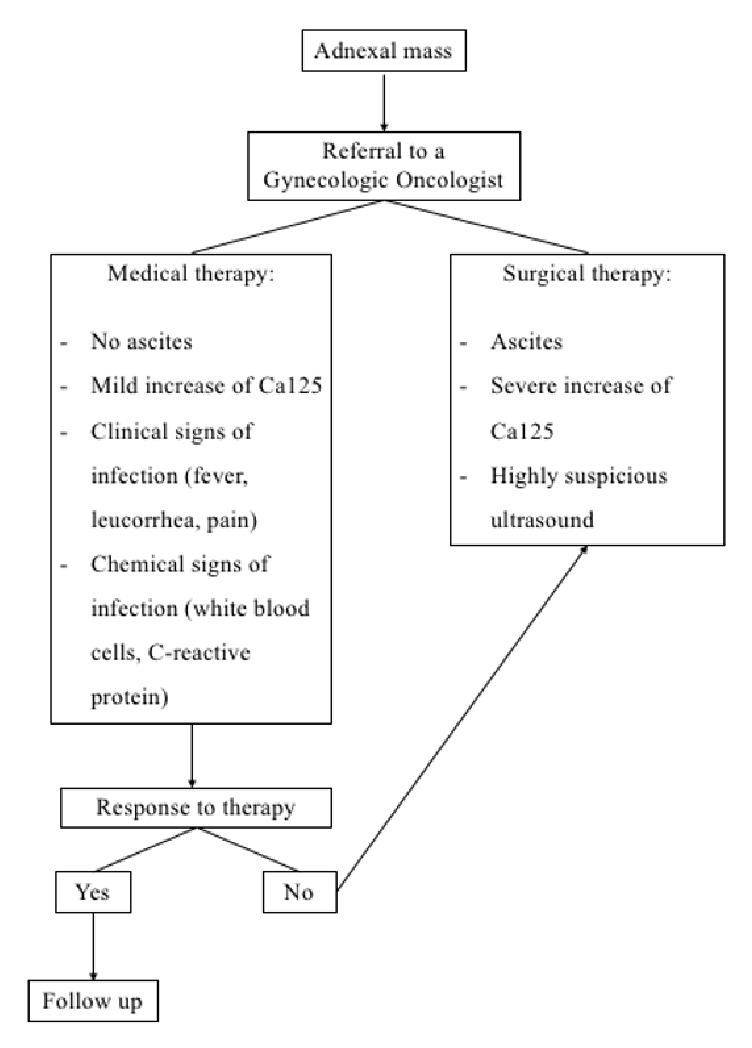
Flowchart.

In our opinion, criteria for choosing medical therapy are mainly related to the absence of ascites, a moderate increase of Ca125, clinical and laboratory signs of infection such as pain, fever, leucorrhoea, CRP, and white blood cells. Conversely, surgery as a first-line treatment may be useful in case of ascites, rapid increase in Ca125, or ultrasound-suspected pelvic mass. In the event of failure to respond to medical treatment, we believe it is mandatory to direct the patient to laparoscopic surgery, as shown in Figure 4.

In case of ascites in combination with high levels of Ca125, laparoscopy with biopsies and cytological examination to confirm any diagnosis should precede laparotomy; diagnostic laparoscopy allows a comprehensive view of the surgical field, but it has its own risks: hemorrhagic, infectious, organ perforation, and aesthetical risks; furthermore, large masses could be broken with pelvic dissemination of suspect material, and this risk is strongly reduced with expert surgeons.

In conclusion, despite guidelines for management of PID, the right timing to switch to surgical therapy is not clear. For this reason, other prospective studies are necessary to properly handle patients with suspected PID and to avoid pitfalls. Moreover, ultrasound diagnosis, biological markers, and laboratory tests have some limitations, particularly with early stage tumors, so future studies should focus on new molecules that may help the early diagnosis. Therefore, the treatment decision should be based on a careful evaluation of various parameters, above all age. Pelvic masses are generally benign in young women, but we believe it is extremely important to refer to a gynecological oncology unit for the correct diagnosis, for a presurgical oncofertility counseling and to obtain the best fertility sparing treatment.
